# Proteomic Analysis of Longitudinal Changes in Blood Pressure

**DOI:** 10.3390/jcm8101585

**Published:** 2019-10-02

**Authors:** Yi-Ting Lin, Tove Fall, Ulf Hammar, Stefan Gustafsson, Erik Ingelsson, Johan Ärnlöv, Lars Lind, Gunnar Engström, Johan Sundström

**Affiliations:** 1 Department of Medical Sciences, Uppsala University, 75236 Uppsala, Sweden; yi-ting.lin@medsci.uu.se (Y.-T.L.); tove.fall@medsci.uu.se (T.F.); ulf.hammar@medsci.uu.se (U.H.); stefan.gustafsson@medsci.uu.se (S.G.); eriking@stanford.edu (E.I.); lars.lind@medsci.uu.se (L.L.); 2 Department of Family Medicine, Kaohsiung Medical University Hospital, Kaohsiung Medical University, 807 Kaohsiung City, Taiwan; 3 Division of Cardiovascular Medicine, Department of Medicine, Stanford University School of Medicine, Stanford, CA 94305, USA; 4 Stanford Cardiovascular Institute, Stanford University, Stanford, CA 94305, USA; 5 Stanford Diabetes Research Center, Stanford University, Stanford, CA 94305, USA; 6 Division of Family Medicine and Primary Care, Department of Neurobiology, Care Science and Society, Karolinska Institutet, 14152 Huddinge, Sweden; johan.arnlov@ki.se; 7 School of Health and Social Studies, Dalarna University, 79131 Falun, Sweden; 8 Department of Clinical Sciences, Cardiovascular Epidemiology, Lund University, 21428 Malmö, Sweden; gunnar.engstrom@med.lu.se; 9 The George Institute for Global Health, University of New South Wales, Sydney 2042, Australia

**Keywords:** proteomics, blood pressure, hypertension, prospective cohort

## Abstract

Hypertension is the leading risk factor for premature death worldwide. The identification of modifiable causes of hypertension remains an imperative task. We aimed to investigate associations between 79 proteins implicated in cardiovascular disease and longitudinal blood pressure (BP) changes in three Swedish prospective cohorts. In a discovery phase, we investigated associations between baseline circulating protein levels assessed with a proximity extension assay and BP stage progression at follow-up 5 years later among persons without BP-lowering drugs at baseline in two independent community-based cohorts from the Prospective Investigation of the Vasculature in Uppsala Seniors study (PIVUS) and the Uppsala Longitudinal Study of Adult Men (ULSAM). We used an independent cohort, the Malmö Diet and Cancer Study (MDC), for replication. The primary outcome of BP stage progression was defined as per the 2017 AHA/ACC (American Heart Association/ American College of Cardiology) Guideline BP categories. We also investigated associations of protein levels with changes in BP on a continuous scale, and meta-analyzed all three cohorts. Levels of renin were associated with BP stage progression with a 5% false discovery rate (FDR) in the ULSAM (*n* = 238) and PIVUS (*n* = 566) cohorts, but we could not replicate this association in the MDC cohort (*n* = 2659). The association in the discovery cohorts was modest, with an odds ratio for BP stage progression over 5 years of 1.33 (95% confidence interval 1.14 to 1.56) per standard deviation of baseline renin. In conclusion, we could not find any novel robust associations with longitudinal BP increase in a proximity extension assay-based proteomics investigation in three cohorts.

## 1. Introduction

Hypertension is a major cause of cardiovascular disease and the leading risk factor for global disease burden [1]. Given the immense public health burden, identification of modifiable causes of hypertension is imperative. Genetic variation is estimated to explain 20–40% of the variation in blood pressure (BP) in the population [2,3]. Numerous physiological alterations have been described in hypertensive individuals, including endothelial dysfunction [4,5], vascular hypertrophy [6,7], cardiac ventricular hypertrophy [8,9], neurohormonal and enhanced sympathetic tone [10,11], abnormalities of renal sodium handling [12,13], reduced fibrinolytic potential [14,15,16], systemic inflammation [17,18,19,20,21,22,23], and enhanced oxidative stress [24]. Some of these factors may be involved in hypertension pathophysiology, but the additional predictive ability of novel biomarkers for hypertension beyond readily available clinical information has been limited [25]. Some circulating biomarkers, such as C-reactive protein (CRP) [19,25,26,27,28,29], plasminogen activator inhibitor-1 [16,25], and aldosterone [30], have been associated with incident hypertension, but results have been conflicting [31,32,33].

Recent technological progress has made the simultaneous measurement of multiple proteins possible, as recently illustrated using a panel of proteins targeted towards cardiovascular disease [34,35]. We hypothesized that these proteins are important also for BP progression. We aimed to explore associations of 92 proteins involved in cardiovascular disease with subsequent BP progression using three population-based cohorts.

## 2. Materials and Methods

### 2.1. Samples

#### 2.1.1. The Prospective Investigation of the Vasculature in Uppsala Seniors Study (PIVUS)

Between 2001 and 2004, all 70-year-old men and women living in Uppsala, Sweden, were eligible for the PIVUS study [36,37]. Among the 2025 individuals who were invited (random selection), 1016 (507 women and 499 men) took part in the investigation (50.1%). A second examination cycle of PIVUS was performed in 2006–2009 when the participants were 75 years old. Of the 964 invited participants, 827 (86%) agreed to the study. In all, the present study sample comprised those 566 individuals who were not under anti-hypertensive treatment at baseline and who provided useful data from the proteomic assay at baseline, data on BP at baseline and at follow-up, and covariates.

#### 2.1.2. Uppsala Longitudinal Study of Adult Men (ULSAM)

The ULSAM study was initiated in 1970. All 50-year-old men born between 1920 and 1924 and living in Uppsala, Sweden were invited to a health survey focusing on identifying cardiovascular risk factors [38,39]. The participants were thereafter invited to examinations at age 60, 70, 77, 82, and 88 years. The present study used the fourth examination cycle as the baseline, when the participants were about 77 years old (1998–2001). Of the 1398 invited men, 838 (60%) participated; 172 were excluded due to lack of plasma for proteomics analysis, and thus 786 were analyzed using the proteomics assay. We used the fifth examination cycle (2003–2005) as a follow-up examination when participants were approximately 82 years old. Among 952 men living in Uppsala, 530 men (56%) participated in this examination. The study sample consisted of 238 men after excluding individuals under antihypertensive treatment, those with missing data on covariates, and those with missing BP measurements at any of the examinations.

#### 2.1.3. The Malmö Diet and Cancer Study (MDC)

The MDC is a prospective population-based study performed between March 1991 and October 1996 designed to elucidate the correlation between diet and other lifestyle factors on the risk of developing cancer [40]. All men born between 1923 and 1945 and women born between 1923 and 1950 living in the city of Malmö were invited to this study. BPs, proteomics analyses and other cardiovascular risk factors were measured in a random subsample of 6103 persons at baseline, which comprised the present study sample. A follow-up examination with measurements of BPs was performed on average 16 years later with the same strategy as the PIVUS and ULSAM. A total of 3734 subjects participated (76% of eligible population). Individuals with antihypertensive treatment at baseline, (*n* = 507), missing BP measurement/s at baseline or follow-up (*n* = 42), and missing protein biomarker measurement/s (*n* = 553) were excluded. The remaining 2659 participants were included in the replication sample [41].

### 2.2. Ethical Considerations

All participants in all cohorts gave written informed consent and the ethics committees of the host universities approved the study protocols (Dnr. 251/90 and 97/329 for the ULSAM; Dnr. 00419, 2005/M-079 for the PIVUS, and LU51/90, LU 2011/537, LU 2012/762 for the MDC). All studies were conducted according to the Declaration of Helsinki.

### 2.3. Baseline and Follow-Up Investigations

After an overnight fast, all participants were examined in the early morning. No medication or smoking was allowed after midnight. The subjects were asked to complete a questionnaire surveying smoking behavior, previous medical history, and current regular medication. Height, weight, and body mass index (weight (kg)/height^2^ (m), BMI) were measured under standardized conditions. Waist circumference was measured at the umbilical level. BP was measured by a calibrated mercury sphygmomanometer. In the ULSAM cohort, a nurse or physician measured BP twice in the right arm to the nearest even number after a 10-min rest in the supine position, and a mean value was calculated. In the PIVUS, BP was measured to the nearest 1 mmHg after at least 30 min of rest in a supine position, and the average of three recordings was used. In the MDC cohort, supine systolic and diastolic BP were measured after 10 minutes of rest using a mercury sphygmomanometer. Fasting blood glucose and lipids were measured by standard techniques [42]. In the PIVUS, serum cystatin C was measured by latex-enhanced reagent (N Latex Cystatin C, Dade Behring, Deerfield, IL, USA) with a Behring BN ProSpec analyzer (Dade Behring). Estimated glomerular filtration rate (eGFR) was calculated from serum cystatin C concentrations (milligrams per liter) by the following formula: y = 77.24 ×cystatin C^−1.2623^ [43]. In the MDC, eGFR was calculated from the MDRD (Modification of Diet in Renal Disease) formula. For patients taking pharmaceutical BP-lowering treatment at follow-up, BP values were imputed by adding 10 mmHg to the systolic BP and 5 mmHg to the diastolic BP [44]. Diabetes mellitus was defined as plasma glucose ≥7.0 mmol/L, or use of oral hypoglycemic agents or insulin. The primary outcome was BP stage progression, defined as per 2017 AHA/ACC Guideline BP categories between baseline and follow-up, as in previous studies [45,46,47], and change in continuous systolic blood pressure (SBP) and diastolic blood pressure (DBP) between baseline and follow-up.

### 2.4. Proteomic Profiling

Venous blood samples were drawn in the morning after an overnight fast and stored at −70 °C. EDTA-preserved plasma samples were assessed with the Proseek Multiplex 96 × 96 proximity extension assay using the Cardiovascular I panel (Olink Bioscience, Uppsala, Sweden) in the three cohorts. The highly specific assay simultaneously measured 92 proteins (Table S1) using two specific antibodies per protein which pairwise bind to each protein, creating a polymerase chain reaction (PCR) sequence from attached oligonucleotide strands when both antibodies are bound to the target protein’s surface. Each sample contains two incubations, one extension, and one detection control used to determine the lower detection limit and normalize the measurements. The values obtained correlate to the concentration of the target protein, without giving absolute concentration values [48]. The resulting relative values were log_2_-transformed for subsequent analysis, and each protein level was normalized by plate by setting the mean to zero and standard deviation to one within each plate and storage time (correction based on the observed values and predicted values from a spline model). Mean intra-assay and interassay coefficients of variation were 8% and 15%, respectively [49]. Normalized protein expression (NPX) values were generated from quantitative PCR quantification cycle (Cq) values, where higher Cq corresponds to lower protein abundance. Cq values (log2 scale) were corrected for technical variation by an interplate control, and lower limits of detection (LOD) were determined through a negative control (NPX = Olink negative control – (△Cqsample – △interplate control)). Values below the LOD were imputed as LOD/2 and normalized for plate. Quality control included removal of proteins with >15% samples below the LOD, and subjects with a high proportion of missing protein values (>5% missing in the PIVUS, and >2% missing in the ULSAM) were excluded. In addition, only proteins passing QC in both discovery cohorts were included. The final data set included 79 proteins; the excluded 13 proteins are listed in Table S1.

### 2.5. Sample Size Estimation

We have used simulated data based on correlations from our own and reported data for a conservative power calculation. We applied a mixed effects ordinal regression model using a Benjamini–Hochberg correction for multiple testing (with a false discovery rate-cutoff of 5%). Under these assumptions, 800 individuals would give us an 80% power to detect proteins with odds ratio of or larger 1.30 (online Supplementary Material).

### 2.6. Statistical Analysis

**Figure 1:**
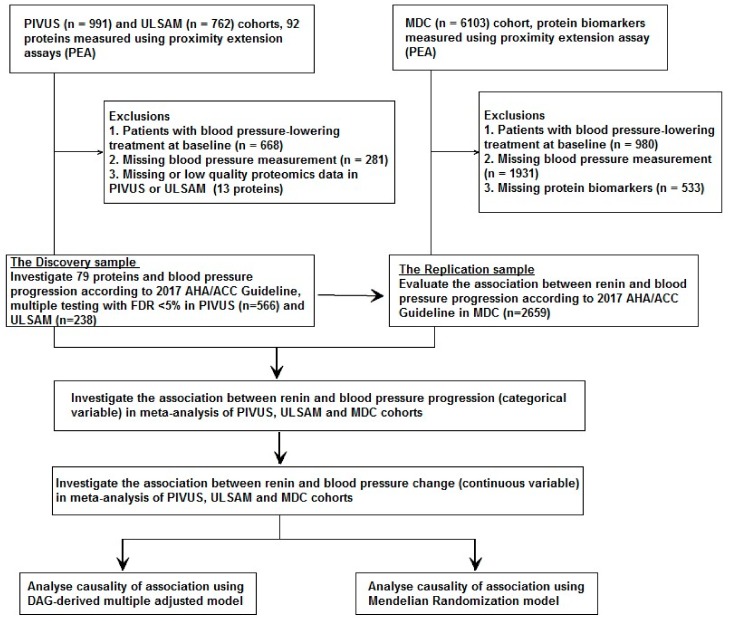
Study flowchart. PIVUS: Prospective Investigation of the Vasculature in Uppsala Seniors study; ULSAM: Uppsala Longitudinal Study of Adult Men; MDC: Malmö Diet and Cancer Study; AHA/ACC: American Heart Association/ American College of Cardiology; FDR: false discovery rate; DAG: DAGitty.

The study design is described in Figure 1. All baseline continuous variables are presented as mean standard deviation and categorical variables as *n* (%). For the first analysis phase, the PIVUS and ULSAM cohorts were used as the discovery sample and the MDC cohort was used for replication. For discovery, the associations between the 79 proteins (each in a separate model) with BP change were investigated using mixed-effects ordered logistic regression models (for BP stage progression) and mixed effects linear regression (for change in continuous SBP and DBP), adjusting for age and sex (fixed effects), and cohort (random intercept). Associations significant at a false discovery rate (FDR) <5% were investigated in the replication sample, adjusting for the same factors. FDR was calculated according to the original version of Benjamini and Hochberg from 1995 [50]. The rationale for this conservative significance threshold was that we wanted to find a reasonable balance between false positive and false negative findings. A nominal *p*-value of <0.05 was considered as a valid replication in the MDC [51,52].

In the next phase, we pre-specified using a one-step individual data meta-analysis of all three cohorts in order to provide the best estimates of the associations. In this dataset, we ranked the proteins by ascending p-value, with bootstrapped confidence intervals around the ranks.

Non-linear associations between proteins and BP stage progression were investigated using restricted cubic splines with four knots.

In the final phase, we sought to investigate the causality of any findings using multivariable-adjusted models and instrumental variables analyses. The choice of variables for the multivariable-adjusted models was based on a causal diagram assisted by the DAGitty, version 2.3 [53] (Figure S1), and the models ultimately included the covariates age, sex, baseline BP, body mass index, waist circumference, smoking, diabetes mellitus, low-density lipoprotein, fasting glucose, estimated glomerular filtration rate, and statin use, all assessed at baseline. We aimed to use Mendelian randomization techniques, to assess potential causal associations between biomarkers and BP stage progression.

All the statistical methods were performed using Stata (version 15, College Station, TX, USA).

## 3. Results

Baseline characteristics of the PIVUS (*n* = 556, mean age 70.2 ± 0.2 years), ULSAM (*n* = 238, mean age 77.6 ± 0.7 years), and MDC (*n* = 2659, mean age 56.2 ± 5.7 years) samples are shown in Table 1. The mean follow-up time was 5.1 ± 0.1 years in the PIVUS, 4.1 ± 0.6 years in the ULSAM, and 16.7 ± 1.5 years in the MDC. During follow-up, 234 (29.1%) of the 804 participants in the two discovery cohorts experienced a ≥1 blood pressure stage progression.

### 3.1. Discovery–Validation Phase

**Figure 2:**
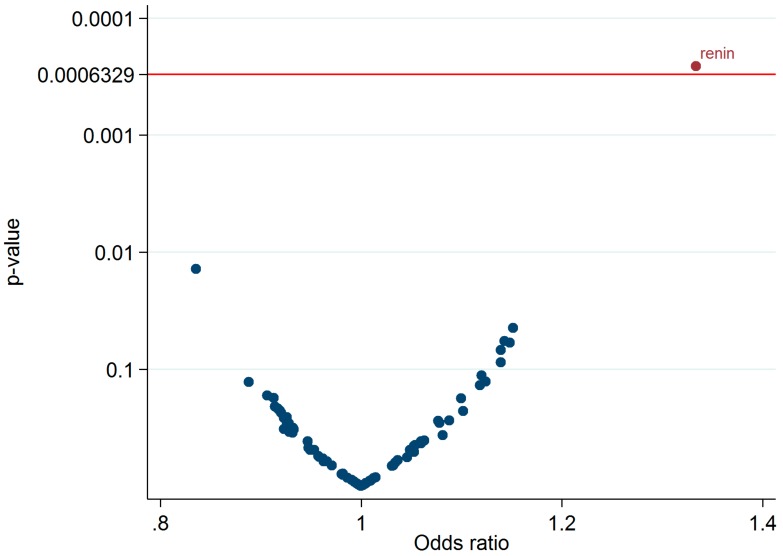
Associations between 79 proteins and blood pressure progression in the Prospective Investigation of the Vasculature in Uppsala Seniors (PIVUS) Study and the Uppsala Longitudinal Study of Adult Men (ULSAM) study. Data are odds ratios (ORs) and 95% confidence intervals (95% CIs).

Relating the 79 proteins to BP stage progression one by one in the discovery sample adjusting for age and sex using a FDR of 5% (corresponding to *p* <6.3E-4; Figure 2), only renin was significantly associated with BP stage progression (OR 1.33, 95% CI 1.14 to 1.56 per SD; Table 2; Table S2). In the replication sample, the association was attenuated and not statistically significant (odds ratio (OR) 1.07, 95% confidence interval (95% CI) 0.97 to 1.19 per SD; Table 2).

### 3.2. Best Estimates Phase

In a meta-analysis of all three cohorts, renin was significantly associated with BP stage progression (OR 1.08, 95% CI 1.01 to 1.15 per SD; Table 2). Investigating associations with change in continuous BPs in all three cohorts, higher baseline renin was associated with higher BP at follow-up (β = 0.69, 95% CI 0.36 to 1.03 for SBP difference, β = 0.43, 95% CI −0.02 to 0.89 for DBP difference; Table 3). Ranking proteins by p-value, renin was the top hit, with a wide bootstrapped confidence interval (1, 95% CI 1 to 17; Figure S2). The cubic spline analysis did not indicate deviation from linearity (Figure S3).

### 3.3. Causality Phase

Although no proteins passed the conservative discovery-validation approach, we attempted to study the potential for a causal but weak association between renin and BP progression. In a meta-analysis of all three cohorts, renin was significantly associated with BP progression in a causal diagram-derived multivariable-adjusted model (OR 1.05, 95% CI 1.01 to 1.08 per SD; Table S3).

We investigated the potential for a Mendelian randomization analysis, but no adequate genetic instruments for renin [54,55] could be identified for instrumental analysis because of power deficit [56,57] (online Supplementary Material).

## 4. Discussion

### 4.1. Principal Observations

In this study, a multiplexed proximity extension assay was used to investigate associations between a large number of circulating cardiovascular disease related proteins and BP progression, in three prospective community samples of 3463 elderly individuals. Using a conservative discovery-validation approach, renin was associated with risk of BP progression in the discovery sample, but not in the replication sample.

### 4.2. Previous Proteomics Studies in Hypertension

Hypertension is a major cardiovascular risk factor with a multifactorial pathogenesis, including genetic and environmental factors. As the technology becomes more sophisticated and available, proteomics analyses may prove useful to help unravel the pathophysiology of hypertension. Urinary proteomics studies have been done, describing urinary nephrin-1 to be associated with salt-sensitive hypertension [58]. Other groups have studied pre-eclampsia- and pregnancy-induced hypertension, which share a number of features with essential hypertension, using urinary proteomics approaches [59].

Studies evaluating the association between circulating proteomics and BP progression are rare. Two previous cross-sectional studies have used mass spectrometry based proteomics to discriminate between hypertensive and normotensive individuals [60,61]. Xu et al. studied serum from 47 patients with essential hypertension and 47 healthy controls, and identified differences between those groups in multiple proteins [60]. Gajjala et al. used a similar design to investigate 118 hypertensive persons and 85 controls [61]. Both studies are limited by their cross-sectional design, small sample sizes and lack of replication.

### 4.3. Previously Studied Proteins Related to Hypertension

Some of the individual proteins that we investigated in this study have previously been evaluated in relation to incident hypertension, such as interleukin-6 [27,31,32] and tumor necrosis factor receptor-2 [32]. In the present study, these biomarkers were not associated with BP progression. Some previously investigated biomarkers (CRP, N-terminal pro-brain natriuretic peptide (NT-pro-BNP)) were not included in the present analysis. Previous observations regarding CRP [19,25,26,27,28,29] and NT-pro-BNP [62,63] are inconsistent.

Both high and low circulating levels of renin may be associated with hypertension. High renin hypertensive individuals are often considered to have vasoconstriction-dependent hypertension [64], with an increased peripheral resistance, whereas hypertension due to primary hyperaldosteronism may be associated with low renin levels [65]. Renin levels in hypertension seem to differ with age [66], and the discovery and validation cohorts differed in age by decades. Evidence for a causal role of renin in hypertension comes from clinical trials of aliskiren, a direct renin inhibitor. Aliskiren has been demonstrated to lower BP [67], but its role in clinical use is unclear.

### 4.4. Strengths and Limitations

Strengths of this study include the longitudinal study design with 5 to 17 years of follow-up, the exhaustive information of study participants, the use of a modern technology proteomics chip based on proximity extension assay that allows for analyses of plasma samples with numerous selected proteins in the same time, and the validation of our results in an independent cohort.

Limitations include differences in age distribution, eGFR formula, and follow-up time in the discovery and validation samples. Plasma renin falls by 17% per decade of age [68,69,70], and elderly people were studied in the PIVUS and ULSAM cohorts and younger people in the MDC cohort. On the other hand, the homogeneity in age and other features within each cohort provide decisive strengths by decreasing confounding. Second, the healthy cohort effect and competing risk from death may be in play because only participants surviving to their second BP measurement at the end of follow up were included; hence individuals with the most rapid BP increases and worst prognosis may have been excluded from the study. Third, the plasma samples had been preserved for more than 10 years, which may have affected the protein levels. However, all samples were stored at −70 °C with only a few freeze–thaw cycles. Previous studies have demonstrated that there is a minor effect of storage time on the protein abundance level [71], so storage time was normalized in the QC process. Further, no adequate genetic instrument could be used for Mendelian randomization analyses. Other limitations include that the protein panel was assembled with candidate proteins previously associated with cardiovascular pathology, with other criteria including concentration limits of the analytes and accessibility of antibodies. Thus, it is not an untargeted proteomics panel; truly untargeted proteomics investigations are still in the future. The scale of the protein assay cannot be converted to absolute concentrations for relevant cutoff values or biomarkers comparison with other studies. Therefore, the application of the proximity extension protein assay in clinical settings merits additional study.

## 5. Conclusions

Exploring a novel proximity extension assay-based proteomics approach, we did not observe any novel replicable associations with longitudinal BP increase.

## Figures and Tables

**Table 1:** Baseline characteristics of participants in the PIVUS, ULSAM, and MDC cohorts.

	PIVUS^†^ (*n* = 566)	ULSAM^††^ (*n* = 238)	MDC^†††^ (*n* = 2659)
Age (years)	70.2 (0.2)	77.6 (0.7)	56.2 (5.7)
Women, *n* (%)	285 (50.4)	0 (0.0)	1640 (61.7)
Smoker, *n* (%)	55 (9.7)	16 (6.7)	498 (18.7)
Systolic blood pressure at baseline (mmHg)	144.9 (21.1)	146.8 (19.3)	136.8 (17.2)
Diastolic blood pressure at baseline (mmHg)	77.0 (9.9)	78.9 (9.3)	85.1 (8.6)
Systolic blood pressure at follow-up (mmHg)	148.8 (19.8)	145.4 (16.5)	147.4 (20.1)
Diastolic blood pressure at follow-up (mmHg)	76.5 (9.5)	81.0 (9.2)	85.2 (10.5)
Body mass index (kg/m^2^)	26.5 (4.0)	25.6 (3.0)	25.2 (3.5)
Waist circumference (cm)	89.5 (11.1)	93.0 (9.6)	81.5 (11.9)
Diabetes mellitus, *n* (%)	35 (6.2)	16 (6.7)	119 (4.5)
Total cholesterol (mmol/L)	5.5 (1.0)	5.5 (1.0)	6.1 (1.1)
Low-density lipoprotein cholesterol (mmol/L)	3.4 (0.9)	3.6 (0.8)	4.1 (1.0)
High-density lipoprotein cholesterol (mmol/L)	1.6 (0.4)	1.4 (0.3)	1.4 (0.4)
Triglycerides (mmol/L)	1.2 (0.6)	1.3 (0.7)	1.2 (0.6)
Fasting glucose (mmol/L)	5.8 (1.3)	5.7 (1.4)	5.5 (0.9)
Estimated glomerular filtration rate (ml/min/1.73 m^2^)	71.6 (14.9)	78.4 (14.5)	83.9 (14.1)
Statin treatment, n (%)	68 (12.0)	27 (11.3)	31 (1.2)
Baseline examination starting year	2001	1998	1991
Anti-hypertensive treatment during follow-up, n (%)	163 (28.8)	65 (27.3)	1354 (50.9)
Length of follow-up (years)	5.1 (0.1)	4.1 (0.6)	16.7 (1.5)

Continuous variables are presented as mean standard deviation and categorical variables as *n* (%). In patients receiving anti-hypertensive drugs used at follow-up, we added 10 mmHg to their systolic blood pressure and 5 mmHg to their diastolic blood pressure. ^†^PIVUS, Prospective Investigation of the Vasculature in Uppsala Seniors. ^††^ULSAM, Uppsala Longitudinal Study in Adult Men. ^†††^MDC, Malmö Diet and Cancer Study.

**Table 2:** Associations of renin and blood pressure progression in the discovery sample (PIVUS and ULSAM) and replication sample (MDC).

	OR (95% CI)	*p*-Value
Discovery sample (PIVUS and ULSAM)	1.33 (1.14 to1.56)	<0.001
Replication sample (MDC)	1.07 (0.97 to 1.19)	0.199
Meta-analysis of all three cohorts	1.08 (1.01 to 1.15)	0.030

OR: odds ratio. Associations of baseline renin normalized protein expression (NPX) value (per SD) with blood pressure stage progression at follow-up examination, using mixed ordered model adjusting for age and sex (fixed effects) and cohort (random effect).

**Table 3:** Associations of renin at baseline with blood pressure change between baseline and follow-up.

	SBP		DBP	
	Linear Mixed Regression		Linear Mixed Regression	
	β-Coefficient	95% CI	β-Coefficient	95% CI
Discovery sample (PIVUS and ULSAM)	1.94	0.65 to 3.23	0.84	0.11 to 1.58
Replication sample (MDC)	−0.52	−1.62 to 0.58	0.48	−0.14 to 1.10
Meta-analysis of all three cohorts	0.69	0.36 to 1.03	0.43	−0.02 to 0.89

SBP: systolic blood pressure; DBP: diastolic blood pressure. β-coefficients express the associations of baseline renin NPX value (per SD) with change in blood pressure between the baseline and follow-up examination, using mixed linear model adjusting for age and sex (fixed effects) and cohort (random effect). CI: confidence interval.

## Supplementary Materials

The following are available online at https://www.mdpi.com/2077-0383/8/10/1585/s1, Figure S1: Proposed causal diagram for the association between renin protein and blood pressure progression, Figure S2: Ranking proteins by *p*-value with bootstrapped confidence intervals around the ranks, Figure S3: Cubic spline analysis of renin protein levels and blood pressure progression in the PIVUS and ULSAM cohorts, Table S1: List of 92 proteins measured by proximity extension assay. The 13 proteins that failed quality control metrics were excluded from the analysis as indicated in the fourth column, Table S2: The association between proteins and blood pressure progression in the PIVUS and ULSAM cohorts, Table S3: The association between renin and blood pressure progression in the PIVUS, ULSAM and MDC cohorts combined.
